# Genome-Wide Quantitative Identification of DNA Differentially Methylated Sites in Arabidopsis Seedlings Growing at Different Water Potential

**DOI:** 10.1371/journal.pone.0059878

**Published:** 2013-04-08

**Authors:** Alejandro C. Colaneri, Alan M. Jones

**Affiliations:** 1 Department of Biology, University of North Carolina at Chapel Hill, Chapel Hill, North Carolina, United States of America; 2 Department of Pharmacology, University of North Carolina at Chapel Hill, Chapel Hill, North Carolina, United States of America; Universidad Miguel Hernández de Elche, Spain

## Abstract

**Background:**

In eukaryotes, the combinatorial usage of cis-regulatory elements enables the assembly of composite genetic switches to integrate multifarious, convergent signals within a single promoter. Plants as sessile organisms, incapable of seeking for optimal conditions, rely on the use of this resource to adapt to changing environments. Emerging evidence suggests that the transcriptional responses of plants to stress are associated with epigenetic processes that govern chromatin accessibility. However, the extent at which specific chromatin modifications contribute to gene regulation has not been assessed.

**Methodology/Principal Findings:**

In the present work, we combined methyl-sensitive-cut counting and RNA-seq to follow the transcriptional and epigenetic response of plants to simulated drought. Comprehensive genome wide evidence supports the notion that the methylome is widely reactive to water potential. The predominant changes in methylomes were loci in the promoters of genes encoding for proteins suited to cope with the environmental challenge.

**Conclusion/Significance:**

These selective changes in the methylome with corresponding changes in gene transcription suggest drought sets in motion an instructive mechanism guiding epigenetic machinery toward specific effectors genes.

## Introduction

Plants possess diverse mechanisms to survive a plethora of environmental conditions. The most common strategy is the adaptive control of genes coping with stress. In eukaryotes, the biochemical activities that regulate the expression of genes are not limited to sequence specific, protein-DNA interactions but also involve the epigenetic control of chromatin accessibility [1] [2]. The local chromatin structure is shaped mainly by the activities of ATP-dependent chromatin remodeling and histone modifying proteins. Additionally, 5-cytosine methylation adds an extra layer of epigenetic information to the primary DNA structure. DNA methylation directly affects protein-DNA interactions, as well as biochemical reactions occurring at the level of the histone coat [3]. Because 5-cytosine methylation is a bimodal property of DNA, many cytosines in the genome, principally those located at CG dinucleotides (CG), have the potential to function as a transcriptional switch [1], [4]. It has been proposed that during the responses of plants to stress, DNA methylation functions as a major switch to control the activity of effector genes [5] [6] [7]. However these “epigenetic marks” also constitute pivotal instructions in the plant developmental program [8] [9] [10] [11]. Importantly, changes in DNA methylation that arise during the life of the plants can be propagated both meiotically and mitotically and hence so will the biological consequences associated with these changes [12]–[14]. Plant organogenesis occurs at both embryonic and postembryonic stages and the whole process is widely exposed to ambient influences. Remarkably, the developmental program can be acclimatized to increase the individual fitness on heterogeneous habitats. It is unclear how much of the phenotypic plasticity shown by plants is controlled at the epigenetic level, but recent experiments with epigenetic recombinant inbred lines (epiRILs) suggested that a wide range of phenotypic possibilities remained hidden by DNA methylation at CG sites [6], [15]. Developmental plasticity is a critical component of the plant response to stress, and the epigenetic contribution to this phenomenom is restricted to the ability of cells to alter their epigenomes in response to environmental stimuli.

The aim of this study was genome-wide identification, cataloging, and interpretation of regulatory loci that are dynamically regulated by methylation in response to stress, particularly drought. We followed changes in methylation in one quarter of all genomic CGs, sampling for drought differentially methylated sites in different sequence environments, including promoters, exons, introns, intergenic regions, transposons and most classes of repeats. We measured the degree of coupling between drought modified gene expression and the identified differentially methylated sites (DMS). We identified a group of drought responsive genes whose activities are potentially regulated by epigenetic mechanisms, while the isolated DMS may mark the regulatory loci. To our knowledge, this is the most comprehensive assessment of a plant methylome dynamics under drought [16]–[18].

Water limitation has become the major threat for crop yield worldwide, thus to understand and manipulate the plasticity of the methylome under drought will direct efforts to increase the efficiency of agricultural systems. This work provides the foundation necessary to accomplish such a goal.

## Results

### Simulated Drought with PEG-infused Agar Media

Seven-day-old seedlings were exposed to controlled and reproducible severities of low water potential (ψ_w_) using PEG-infused agar plates as described by Verslues [19]. Plants were collected immediately after the end of treatment and stored at −80°C until a total of four independent experiments were performed.

**Figure 1:**
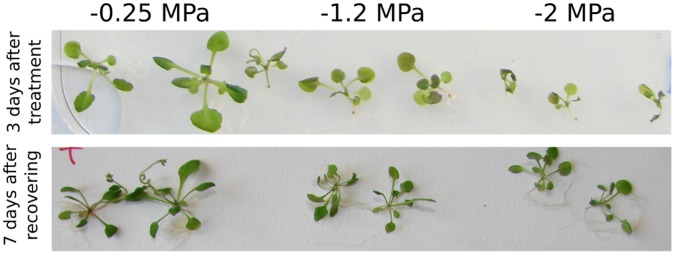
Growth phenotype of Arabidopsis Col 0 exposed to different water potential. **Upper panel**: plants were first grown under constant light on plates containing ¼ MS media with a water potential of −0.25 MPa (ample water control condition). Seven-day-old seedlings were transplanted to new plates that were infused with PEG to obtain the indicated (−0.25, −1.2, and −2 MPa) water potentials as described in Methods. Representative seedlings are shown. **Lower panel**: After the 3 days treatment, seedlings were transferred to fresh ¼ MS media plates with water potential of −0.25 MPa. After 7 day, representative seedlings are shown.

Figure 1 upper panel, shows the phenotypes of seedlings after 3 days on control plates (ψ_w_ = −0.25) or treatment plates (ψ_w_ = −1.2 and ψ_w_ = −2). Obvious differences were observed between plants grown at the different ψ_w_. This stress phenotype was reversible since seedlings recovered after transfer to −0.25 MPa (Figure 1 lower panel), indicating that tissue samples were collected from living seedlings.

### Methyl Sensitive Cut Counting Profiles: Treatment Reproducibility and Induced Differences

Genomic DNA samples, isolated from 16 individual pools of treated Arabidopsis seedlings, were digested with four different methylation sensitive restriction enzymes (MSRE): HpaII, AciI, HypCH4IV and Hinp1I. Changes in the level of methylation were detected by comparing digestion frequencies at 634,440 sites. These loci are an even sample of the total CG sites throughout the genome, (Figure S1). Digestion frequencies were computed through methyl sensitive cut counting (MSCC) [20]–[22]. Briefly, if a particular site is not methylated, the MSRE digests the DNA producing terminals with a 5′-CG overhang. Double-stranded DNA adapters were ligated and then digestion with EcoP15I releases 27-bp fragments from the terminals. We designated these sequences immediately flanking the CG as “CG tags”. The abundance of any individual tag in a CG tag library (digestion frequency) is inversely proportional to the methylation state of the digested site. Accordingly, a poorly represented tag is: **1)** the consequence of local low sequencing coverage, **2)** high rate of methylation at the digested site or, **3)** both. However, the use of MSCC to quantify absolute levels of methylation is limited by the existence of biases that are site specific, i.e. sites in the genome, which tend to be harder to digest in a manner that is independent of its state of methylation [21], [22]. It has been shown that these site-specific biases are systematic and reproducible among replicates [22]. Since we contrasted the digestion frequencies in a site-by-site basis, these systematic biases are expected to cancel out. In addition, the effect of random errors in these contrasts, is weighed in the denominator of the equation used whenever T statistics were computed. Thus, we statistically infer that differences in the digestion frequencies are mainly attributable to differences in methylation levels.

**Figure 2:**
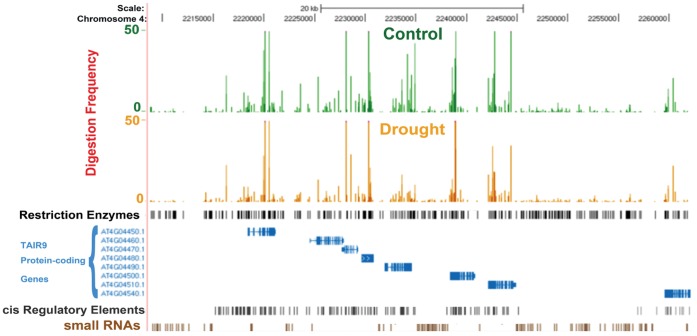
Comparison of methyl sensitive cut counting profiles in simulated drought and ample water. Digestion frequencies maps: Reads were mapped to the Arabidopsis reference genome (TAIR 7 release), and visualized in the UCSC genome browser. The local view shows the average digestion frequencies (average number of reads) associated to each one of the surveyed CG sites (track 3) included in the indicated interval of the chromosome 4. Sites overlapping 5′ region of genes were hypersensitive to the four restriction enzymes, indicating a predominance of un-methylated sites. Oppositely, methylated sites located at intergenic regions were consistently mapped with a low number of reads, suggesting high level of methylation. Description of the UCSC genome browser panel: Track 1- average digestion frequency profiles, 4 replicates, −0.25 MPa treatment; Track 2- average digestion frequency profiles, 4 replicates, −2 MPa treatment; Track 3- surveyed CG sites; Track 4- all CG sites; Track 5, TAIR 7 gene description; Track 6- AGRIS cis-regulatory elements.

Figure 2 shows a comparison of the average digestion profiles recorded for 349 CGs found in a 54,000 bp region of chromosome 4. Sites that were found hypersensitive to the enzymatic digestion segregated from those that were resistant. For the most part, highly digested sites were found in the 5′ regions of genes while poorly digested ones were located in intergenic regions. A juxtaposition of the digestion profiles with a track that outlines the distribution of cis-regulatory elements displayed the same trend. While these results provide cogent evidence on the effectiveness of MSCC to capture quantitative information about the distribution of methylation in the genome, the focus of the present study was to determine differences produced by the treatment rather than to profile the absolute level of methylation along the genome.

**Figure 3:**
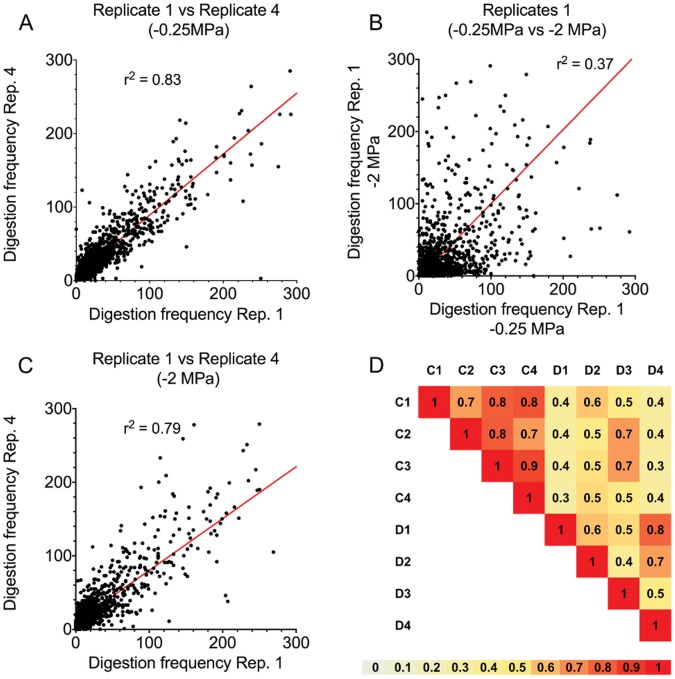
Genome wide impact of simulated drought in the Arabidopsis methylome. The coefficient of determination (r^2^) was used to quantify similitudes in the distribution of methylation among different samples. For the genome-wide estimation, 1,200 CG sites were randomly selected. **A**) scatter plot comparing the digestion frequencies in two replicates representing the ample water condition. **B**) scatter plot comparing digestion frequencies of samples treated at different water potential but in the same experimental replication. **C**) scatter plot comparing the digestion frequencies in two replicates representing the drought condition (−2 MPa). **D**) A summary of all vs. all pairwise comparisons. C = Control; # = replicate number; D = simulated drought, numbers are the correlation coefficients. Scale below represents heat map for similarity.

Out of the 634,009 CG sites surveyed, 547,427 were represented by at least one read in each library. To increase confidence, we compared the digestion frequencies of sites that had a minimum of 20 reads in any of the prepared libraries yielding a set of 109,458 sites between the 2 methylomes (control vs. PEG-treatment) with four biological replicates per treatment. Figure 3 illustrates the genome wide impact of drought on the Arabidopsis methylome. A significant (r^2^ = 0.83, r^2^ = 0.79) linear relationship results when two replicates within the same class of treatment (−0.25 MPa or −2 MPa) are compared indicating reproducibility within treatments and a consistent distribution of methylation among biological replicates. However, no correlation in digestion frequencies between treatments were found (r^2^ = 0.35), indicating differences between the methylomes of control vs. PEG-treated plants (Figure 3B). Figure 3D shows the r^2^ values for the all-by-all comparison confirming methylome reproducibility within treatments and major differences between treatments.

### Identification of CG Dinucleotides whose Methylation Status is Modified during the Treatment

**Figure 4:**
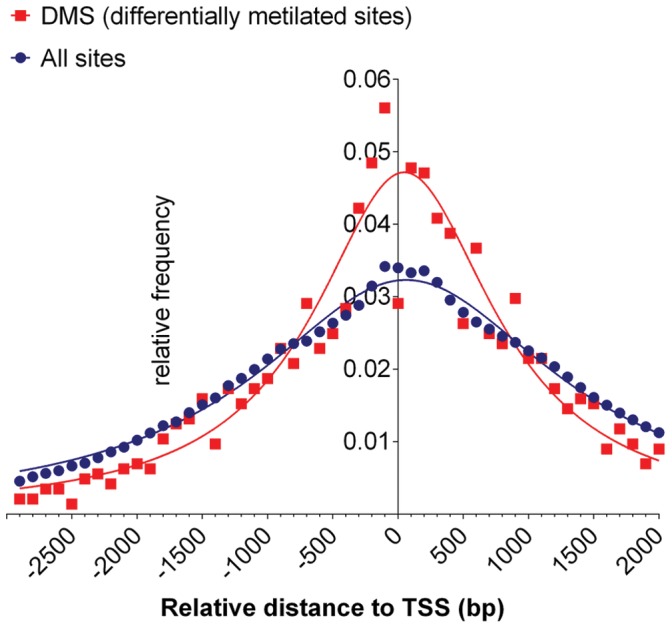
Distribution of differentially methylated sites (DMS) in relation to the transcription start sites. Distribution of DMS in the Arabidopsis genome: Arabidopsis genes were aligned relative to their transcription start sites. The Y-axis represents the relative frequency of DMS computed for 100-bp intervals along the X-axis (red curve) or the relative frequency of all restriction sites used in this study (blue curve). The X-axis represents the relative distances to the TSS.

For the identification of sites whose methylation levels varied consistently between the two treatments, we performed Welch t tests. The null hypothesis was stated as no difference between the averaged replicates. When the Welch t test detected a difference, means were sorted by size and the differentially methylated sites (DMS) were classified as either “methylated” or “demethylated”. We detected 10,862 DMS with a false discovery rate (FDR) of 0.05, which comprised 9,898 events of methylation and 964 events of demethylation. To increase confidence, we set the FDR to 0.01 and assembled 1,552 DMS for in-depth analyses, Data Set S2. Unlike the distribution of DMS associated with epimutation, which are found primarily within exons and introns [23], [24], the drought-associated DMS pattern shown here centered at the transcriptional start sites (TSS). The relative abundance of DMS significantly exceeds the relative abundance of all sites found at a distance of 500 bases from the TSS, (Figure 4). The possibility that these relative distributions differ by chance was subjected to a test through 100 simulations. Each time the relative distribution of DMS exceeded the relative distribution of all restriction sites located a 500 bp from the TSS. This enrichment (p-value <0.01) suggests “promoter-hot-spots” allocating environmental-responsive and epigenetic-controlled DNA-regulatory-elements.

While changes in methylation state occurred in both directions, the vast majority of the DMS consisted of CG that increased methylation during the treatment.

### Gene Annotation Enrichment Analysis

Genes whose TSS were the closest to a DMS were assigned to two groups: the methylated promoter set (“met data set”), which contained 7,419 genes associated with at least one event of methylation and the un-methylated promoter set “unmet data set” containing 934 genes associated with at least one event of de-methylation (FDR = 0.05) (Data Set S2 and S5). These two lists were translated into functional profiles aimed to provide insights into the biological mechanism based on the gene ontology database (GO) in Table 1. The set of overrepresented GO terms matched the most distinctive attributes that characterize genes involved in the physiological and biochemical response of plants to stress, in particular water and osmotic stress [25]–[27].

**Table 1:** GO enrichment analysis results.

GO term Description	P-value	FDR q-value	^1^Enrichment (N, B, n, b)
**biological process for the “met data set”**			
response to stimulus *	2.52E−13	4.83E−10	**1.14** (24410,5797,6216,1688)
response to chemical stimulus *	2.52E−12	3.22E−09	**1.19** (24410,3325,6216,1011)
response to abiotic stimulus *	2.31E−11	1.48E−08	**1.23** (24410,2269,6216,711)
response to abscisic acid stimulus	4.43E−07	7.70E−05	**1.40** (24410,484,6216,172)
organ development	6.90E−06	6.44E−04	**1.41** (24410,363,6216,130)
drug transmembrane transport	9.11E−06	8.11E−04	**2.03** (24410,62,6216,32)
response to ethylene stimulus	1.04E−05	9.06E−04	**1.44** (24410,300,6216,110)
response to cadmium ion	1.17E−05	9.95E−04	**1.38** (24410,393,6216,138)
response to osmotic stress	2.17E−05	1.51E−03	**1.27** (24410,678,6216,220)
response to stress	4.27E−05	2.55E−03	**1.11** (24410,3386,6216,956)
response to salt stress	5.42E−05	3.00E−03	**1.27** (24410,638,6216,206)
organ morphogenesis	1.10E−04	5.76E−03	**1.38** (24410,295,6216,104)
carbohydrate metabolic process	1.32E−04	6.74E−03	**1.15** (24410,1680,6216,492)
metal ion transport	1.86E−04	9.23E−03	**1.28** (24410,507,6216,165)
regulation of anion channel activity	2.03E−04	9.34E−03	**2.33** (24410,27,6216,16)
auxin polar transport	2.81E−04	1.16E−02	**1.72** (24410,80,6216,35)
response to red or far red light	3.06E−04	1.23E−02	**1.33** (24410,336,6216,114)
monocarboxylic acid metabolic process	4.55E−04	1.68E−02	**1.17** (24410,1074,6216,321)
chloroplast organization	5.09E−04	1.85E−02	**1.44** (24410,186,6216,68)
protein targeting to membrane	5.27E−04	1.89E−02	**1.31** (24410,354,6216,118)
cellular nitrogen compound catabolic process	5.53E−04	1.96E−02	**1.39** (24410,226,6216,80)
response to auxin stimulus	5.94E−04	2.07E−02	**1.31** (24410,355,6216,118)
cellular protein modification process	6.02E−04	2.08E−02	**1.12** (24410,2090,6216,595)
auxin homeostasis	7.38E−04	2.30E−02	**2.54** (24410,17,6216,11)
root hair cell differentiation	7.82E−04	2.39E−02	**1.52** (24410,124,6216,48)
xylem development	8.42E−04	2.50E−02	**1.71** (24410,69,6216,30)
negative regulation of transcription, DNA-dependent	9.29E−04	2.65E−02	**1.31** (24410,321,6216,107)
regulation of plant-type hypersensitive response	9.59E−04	2.70E−02	**1.30** (24410,335,6216,111)
**molecular function for the “met data set”**			
acid phosphatase activity	1.53E−07	6.87E−05	**2.84** (24410,29,6216,21)
transmembrane transporter activity	5.45E−07	1.53E−04	**1.29** (24410,873,6216,286)
secondary active transmembrane transporter activity	1.93E−06	3.61E−04	**1.51** (24410,262,6216,101)
flavin adenine dinucleotide binding	6.93E−06	9.74E−04	**1.66** (24410,144,6216,61)
ATP binding	6.13E−05	5.30E−03	**1.15** (24410,1818,6216,533)
secondary active sulfate transmembrane transporter activity	6.93E−05	5.76E−03	**3.93** (24410,7,6216,7)
heme binding	2.50E−04	1.52E−02	**1.33** (24410,348,6216,118)
antiporter activity	3.56E−04	1.86E−02	**1.53** (24410,136,6216,53)
ATPase activity, coupled to transmembrane movement of substances	4.47E−04	2.19E−02	**1.49** (24410,153,6216,58)
inorganic cation transmembrane transporter activity	5.83E−04	2.62E−02	**1.37** (24410,243,6216,85)
UDP-N-acetylmuramate dehydrogenase activity	5.99E−04	2.64E−02	**1.92** (24410,45,6216,22)
water transmembrane transporter activity	8.42E−04	3.50E−02	**2.32** (24410,22,6216,13)
water channel activity	8.42E−04	3.57E−02	**2.32** (24410,22,6216,13)
**biological proccess for the “unmet data set”**			
response to stimulus *	1.46E−06	1.11E−03	**1.31** (24113,5653,760,234)
raffinose family oligosaccharide biosynthetic process	6.12E−07	5.79E−04	**22.66** (24113,7,760,5)
respiratory burst involved in defense response	3.24E−05	9.45E−03	**3.85** (24113,107,760,13)
regulation of meristem structural organization	4.85E−05	1.22E−02	**11.33** (24113,14,760,5)
negative regulation of gibberellic acid mediated signaling pathway	6.19E−05	1.38E−02	**15.86** (24113,8,760,4)
monocarboxylic acid metabolic process	8.00E−05	1.59E−02	**1.70** (24113,1044,760,56)
hyperosmotic salinity response	1.39E−04	2.39E−02	**3.35** (24113,123,760,13)
carboxylic acid biosynthetic process	1.43E−04	2.26E−02	**1.71** (24113,945,760,51)
sesquiterpenoid biosynthetic process	4.32E−04	4.96E−02	**5.95** (24113,32,760,6)
jasmonic acid mediated signaling pathway	5.56E−04	5.54E−02	**2.41** (24113,237,760,18)
response to water deprivation	6.68E−04	6.17E−02	**2.15** (24113,324,760,22)
response to auxin stimulus	6.82E−04	6.00E−02	**2.11** (24113,346,760,23)
response to chitin	6.89E−04	5.93E−02	**2.07** (24113,368,760,24)
anatomical structure development	7.05E−04	5.93E−02	**1.53** (24113,1268,760,61)
regulation of defense response	8.42E−04	6.50E−02	**1.92** (24113,463,760,28)
regulation of cellular biosynthetic process	9.22E−04	6.71E−02	**1.37** (24113,2249,760,97)
attachment of spindle microtubules to kinetochore	9.92E−04	6.71E−02	**31.73** (24113,2,760,2)
**molecular function for the “unmet data set”**			
binding	2.05E−04	4.53E−01	**1.16** (24113,9528,760,348)
nucleic acid binding transcription factor activity	3.10E−04	3.44E−01	**1.52** (24113,1459,760,70)
sequence-specific DNA binding transcription factor activity	3.10E−04	2.29E−01	**1.52** (24113,1459,760,70)
catalytic activity	4.45E−04	2.47E−01	**1.18** (24113,7647,760,284)

Table 1 GO enrichments from the directed acyclic graph structure. The table includes terms with the lowest p values from the top of the hierarchy (root nodes in the directed acyclic graph labeled with starts) and all terminals nodes describing the most specific biological processes that are significantly enriched. ^1^Enrichment indicated in bold is the fold increase calculated per the following: **Enrichment = (b/n)/(B/N).**

N - is the total number of genes associated to any GO term.

B - is the total number of genes associated with a specific GO term.

n - is the number of genes in the target set.

b - is the number of genes in the intersection.

### The Methylated Data Set

As indicated by the top terms in Table 1, this set contained genes involved in the response to stimulus, including chemical and abiotic signals. Among terms assigned to the terminals nodes, “response to absicisic acid stimulus” appeared with the lowest p value. One quarter of the GO terms for biological process and 70% of the GO terms for molecular functions were related to membrane transport including water transport. The GO term “water channel activity” included 13 out of the total 22 aquaporin genes in the reference list. Finally, 20% of the GO terms describing biological proccess in the “met data set” contained genes involved in organ development and morphogenesis, mainly affecting postembrionic root develpment.

### The Un-methylated Data Set

The functional profile for this set of genes was enriched in terms related to hyperosmotic salinity response and response to water deprivation. Most of the genes associated with water deprivation were transcription factors, such as MYBR1, ZFHD1, ABF2 and HSF4. These four transcription factors regulate a constellation of abiotic stress-inducible genes [28]–[30] [31]. MYBR1, ZHFD1 and ABF2 overexpressing plants have enhanced tolerance to water and salt stress while the loss-of-function mutants shows the opposite phenotype. MYBR1 modulates the antagonistic effects of jasmonate and abscisic acid in the drought response [32]. Genes involved in the negative regulation of gibberellic acid-mediated signalling pathway, including RGA1, RGL2, RGL3, GID1B and RGL1, were de-methylated by simulated drought. These genes are important in salt and osmotic stress responses [33]. Also enriched was the GO term “sesquiterpenoid biosynthetic process” which defined here a set of genes known to be important for drought-induced biosynthesis of ABA including AAO2, NCED3 and ABA1. AAO2 gene encodes one of four aldehyde oxidases that catalyze the last step of ABA biosynthesis in Arabidopsis [34]. NCED3 is a key enzyme in the biosynthesis of abscisic acid. NCED3 is regulated in response to drought and salinity and overexpression of this enzyme increases the endogenous ABA levels thus triggering transcription of drought and ABA inducible genes [35]. ABA1 catalyzes the reaction that initiates the biosynthesis of ABA and mutants plants lacking ABA1 are severely impaired in stress induced ABA production [36].

### Genes that Change their Expression during Simulated Drought

The transcriptome of seedlings exposed to −0.25 MPa vs. −2 MPa water pressure was determined by RNA-seq analysis. RNA samples used in the production of RNA-seq libraries, were derived from the same collection of replicates from which DNA was extracted for methylation measurement. A total of 1,077 genes were identified having a fold-change larger than 1.5 and a FDR <0.05. From those, 600 genes were down regulated and 477 were up regulated, Data Set S3. Results obtained from gene ontology enrichment analysis are presented in Data Set S4. There is a noticeable difference in the kind of biological proccess asociated with these two lists. Most of the down-regulated genes were associated with photosynthetic activity, light perception, plastid organization, photosystem assembly and a diversity of biosynthetic pathways. In contrast, the set of genes with increased steady-state levels of mRNA included mostly biological processes associated with stress, in particular water and osmotic stress.

Out of the 1,078 genes found to change the level of expression as consequence of the PEG treatment, 316 had a modified level of methylation in the proximities of their transcriptional start sites; 167 and 149 were down and up regulated genes, respectively. The association between DMS in the proximities of a gene TSS and changes of the steady-state level of mRNA of the encoding gene, was measured with an odds ratio. The scored ratio was 1.67, indicating that, at least at the genomic scale, there is a poor or not association between these two measured outcomes.

## Discussion

We used methyl sensitive cut counting [22] and RNA-seq to detect differences in the methylomes and transcriptomes of 7-day-old seedlings grown 3 days at two water potentials, −2 MPa and −0.25 MPa (simulated drought and well-watered control, respectively). Incorporation of polyethylene glycol 8,000 in the growth substrate decreases the water potential and induces osmotic stress. This imposed osmotic-force withdraws water from both the apoplast and the cytoplasm, resulting in cytorrhysis, mimicking the effects of soil desiccation. This simulated drought system offers several advantages. First, the water potential at which plants are exposed remains constant during the treatment and transpiration is minimal. Thus, the severity of the imposed stress is accurately controlled, which is critical to obtain reliable results between experimental replicates [19]. Second, the biochemical and phenotypic responses of *Arabidopsis thaliana* growing under this drought-simulated system were already characterized, providing a biological context to place our new findings, [19], [37].

During the first 24 hours of treatment, the seedlings undergo rapid dehydration triggering a concerted set of responses [19], [38]. There is a rapid induction of stress response genes, both ABA dependent and ABA independent, followed by active accumulation of compatible solutes such as proline. Proline concentration increases steadily to reach a plateau by the end of the treatment [19], [38], [39]. The final concentration is linearly controlled by the negative potential of the substrate. Measurements made at water potentials in the range of −1.2 to −2 MPa, 96 hours after starting treatment, showed increases in total proline content of 44 times or more [19], [39]. Proline prevents cellular damage caused by ROS or unbalance redox status, but also contribute to osmotic adjustment. Osmotic adjustment occurred within 72 h of growth, on a substrate with ψ_w_ of −1.2 MPa, accumulates half of osmolytes needed to fully compensate the water pressure gradient. At ψ_w_ of −1.6 or lower, the accumulation of inorganic and organic osmolytes is insufficient to offset the dehydration and likely the seedling experienced loos of turgor [19]. The water potential in the agar media also affects how plants grow. Primary root elongation is reduced when ψ_w_ in the substrate is below −0.5 MPa. The root elongation rate increases steadily to reach a plateau by the third day of treatment. At ψ_w_ of −1.2 MPa, the total elongation of primary root is half of that achieved by seedlings growing in control medium, [37]. The progressive inhibition of root growth associated with the drop in water potential follows an exponential decay. There is a rapid decrease in the elongation rates until the water potential falls below −1 MPa, from when the growth appears less affected [40]. Differently, growth inhibition is much more drastic in the shoot; whereas the root still upholds some growth at ψ_w_ lower than −1.5 MPa, the shoot stops growing at higher potentials [40]. This differential growth increases the root/shoot ratio and constitutes a general response of plants to low water potential [37], [41].

Thus seedlings that have been transferred to PEG-infused agar plates equilibrate with the water potential of the substrate over the time, and on the third day of treatment, mechanisms of dehydration avoidance and tolerance are already underway. We assessed the extent to which the Arabidopsis methylome responds to simulated drought. We further explored the possibility that certain genomic environments are preferentially selected for differential methylation during the treatments. Consistent with its cis-acting gene regulatory activities, sequences proximal to the TSS showed enrichment in DMS. The prevalent mode of differential methylation during the growth at low water potential was hypermethylation. Interestingly genes affected by hypermethylation were widespread in the genome but functional profiles derived from them showed enrichments in activities related to stress responses.

The differences in methylation could in part be explained by epigenetic variation between corresponding tissues and in part by changes in the proportions of different tissues or cell types, e.g. an increased root/shoot ratio for the plants grown at lower water potential [37]. However parsing the constituent sources of this epigenetic variation will require advances techniques for sample collection, such as tissue micro-dissection or cell sorting. This type of analysis, although highly informative, is beyond the objectives of the present work.

Do the observed DNA methylation changes modulate the activity of genes in response to drought? When we compared the functional profiles obtained from our lists of genes “differentially expressed” with genes “differentially methylated”, we found similar predicted biological functions. Out of the 1,077 differentially expressed genes, 321 contained differentially methylated sites in their promoters. A functional characterization of this set revealed that the highest enrichment occurred with genes involved in ionic homeostasis and water transport.

We acknowledge that the confidence in our functional analysis is limited by the quality of the GO database, and our primary concern resided in the kind of evidence supporting the functional annotations of these genes. We inspected the annotation details of each gene included in the most relevant enriched categories and found a preponderance of hand curated and experimental data supporting the assignments. For example, in the enriched GO term “water deprivation” we found 22 genes where 14 of them were annotated based on mutant phenotypes and expression patterns measured for those particular genes under water stress. The other 8 genes were annotated based on a meta-analysis of genome-wide experimental data.

A surprising observation was the lack of association between changes in methylation and changes in gene expression. This may in part be due to thresholds selection and detection limitations. For example, among the 22 Arabidopsis aquaporin genes, 13 were methylated in their promoter, but only 4 of them had associated decreases in steady-state mRNA as detected by RNA-seq, although expression of all 22 genes were reported to be downregulated during drought or salt when quantitative real time PCR or macroarray with gene specific tags were used to evaluate their transcription level [42], [43].

Importantly, the quantification of digestion frequencies in whole seedlings gauges the average DNA methylation attained by the relative contribution of the different cell types to the pooled genomes. Since for each cell, DNA methylation is a binary property, the changes detected in a pool of genomes depend only on the number of cells varying between the two possible epigenetic states. On the other hand, the observed changes in mRNA levels reflect not only the amount of cells but also the magnitude of the mRNA changes in each cell. Thus, a large increase in the expression of a gene in a rare cell type may mask the repressive effects of DNA methylation in a most common cell type.

In the present study, we discovered an epigenetic response primarily targeted to stress-response genes, prompting the hypothesis that drought sets in motion an instructive mechanism guiding epigenetic machinery toward specific effectors genes. Whether these methylation changes bring out adaptive advantages for drought tolerance or avoidance, remain to be elucidated.

Part of differential methylation marks may function as a record of exposure, placing the chromatin in an “alert state”, thus allowing the plants to respond faster and more effectively to a subsequent event of drought. Evidence in favor of this idea have been recently published; histone dependent epigenetic marks are introduced into the chromatin as part of a transcriptional training mechanism, which operates during multiple exposures to drought, [44].

## Methods

### Simulated-drought Treatment

Seeds of *Arabidopsis thaliana* ecotype Columbia were surface sterilized with 70% ethanol solution for 10 min, and 95% ethanol solution by another 10 min followed by 2 days stratification at 4°C on plates. Plates (120 mm×120 mm, Sigma Aldrich Cat # Z617679-240EA) were made with ¼ Murashige and Skoog (Calsson Labs, Cat # MSP01) and 1.5% phytoagar (RPI Corp. Cat # A20300-1000.0). Plates were moved to a growth chamber under constant light conditions (21°C, 60 umole m^−2^ s^−1^). Seven-day-old seedlings were transplanted to plates with lowered water potential (−1.2 MPa or −2 MPa) or control plates with the same water potential (−0.25 MPa) prepared exactly as described by [19]. Briefly, water potential was lowered by infusing the agar plates with various concentrations of polyethylene glycol (PEG). After 3 day of growth, seedlings were harvested in parallel and clumps containing aproximately 100 mg of tissue were aliquoted in sterilize 2-ml screw cap tubes (Olympus Plastic, Cat # 21–254), containing two 3.2-mm, stainless-steel beads (Biospec products, Cat # 11079132 ss). Samples were flash frozen in liquid nitrogen before storage at −80°C. Four independent experiments were performed on different days. A detailed protocol for the PEG-treatment is provided as Data Set S6.

### Nucleic Acid Extraction

Samples stored at −80°C were immersed in liquid nitrogen for 1 min. Tissue was pulverized by 3 cycles (20 s at 6.5 m/s speed each) in a Fastprep 24™ instrument (MP Biomedicals, SKU # 116004500). Samples were flash frozen in nitrogen liquid at the end of each cycle. Total genomic DNA or total RNA were extracted from the pulverized material using the DNeasy Plant Mini Kit ™ or the RNeasy Mini Kit, respectively (QIAGEN, Cat # 69104 and Cat # 74104), following the manufacturer’s instructions. Genomic DNA and total RNA concentration and purity was determined with a Nanodrop 2000 spectrophotometer (Thermo Scientific). Genomic DNA integrity was evaluated by visualization after electrophoresis in 0.8% agarose gel containing ethidium bromide. Total RNA integrity was determined using a Bioanalyzer Chip RNA 7500 series II (Agilent).

### Transcriptomic Analaysis

Six RNA samples (2 water potential×3 replicates) were used to prepare 6 barcoded non-directional Illumina RNA-Seq libraries using the TruSeq RNA Kit (ILLUMINA Cat # RS-930-2005). The quality of each library was analyzed with a Bioanalyzer Chip DNA 1000 series II (Agilent). Each library had an average insert size of 200 bp, and an average concentration of 17 ng/ul. Equal amounts of each library were mixed before sequencing. Sequences of 50 base pairs were produced with Illumina HiSeq 2000. Sequences with different barcodes representing the transcriptome for different replicates were sorted in different FASTQ files. The Tophat tool from the Galaxy mirror supported at UNC (https://galaxy.its.unc.edu/) was used to map RNA-seq reads contained in these FASTQ files to the TAIR 10 genome release. A total of 58 million 50-bp reads were made with an average of 9.7 million reads per library, an appropriate density to perform quantitative analysis of gene expression. Each BAM dataset produced by Tophat was imported into the PARTEK genomic suite 6.3 software (Partek Inc. 2008) and RPKM (reads per kilobase of exon model per million mapped reads) were calculated for TAIR10 Arabidopsis transcripts. Differential expression analysis between groups (−0.25 MPa vs −2 MPa treatments) was performed using ANOVA and differentially expressed transcripts were selected by applying fold-change cutoff of 1.5 and p-value cutoff of 0.05 (Data Set S3).

### Genome Wide Methylation Analysis

Eight DNA samples (2 treatments×4 replicates) were used to prepare the MSCC libraries following most of the guidelines and methods published in [22], with the difference that 2 ug of gDNA was used as starting material and 4 different barcoded adapters were used in the final ligation reaction prior to the amplification of the libraries (Data Set S6). Each barcode represented a unique experimental replicate. Libraries from the four replicates representing the −0.25 MPa treatment were combined in a single library called “control” and the 4 libraries representing the replicates for the −2 MPa treatment were combined in another single library called “drought”. These two libraries were sequenced separately using an Illumina HiSeq 2000. We generated an average of 32 million sequence reads per library (control or drought) each 50 bp in length. According to the design of our libraries, the first 27 characters of each sequence are the CG-tags. The next three letters describe the barcodes followed immediately by the adapter B sequence. An in house Perl script was used to sort the sequeces according to their bar codes previous to the aligment. Sorted reads were aligned to the TAIR 7 Arabidopsis genome release using MOM [45], allowing one mismatch. Each CG was identified by their forward or reverse tags, however for the purpose of this analysis the counts in both tags were collapsed in a single number representing the digestion frequency at the particular CG site. The distribution of reads among the 8 different libraries is shown in Data Set S6. Since the total number of sequences resulted not equally distributed among the different replicates, reads were normalized at each identified CpG by a factor, proportional to the total number of reads from each particular library, Data Set S2. Comparison between treatments and analysis for differences between means of control and drought groups were performed using T test. The threshold for statistical significance was FDR <0.05 or FDR <0.01.

### Gene Ontology (GO) Enrichment Analysis

Functional profiles were obtained using Gorilla [46]. Data Set S5 contains all the gene lists used in these analyses. Each list consisted of official gene symbols representing genes that were found differentially methylated or differentially expressed between treatments. To build a background list, all the AGI codes representing the Arabidopsis genes were obtained from the TAIR web site. AGI codes were converted to official gene symbols using the gene ID conversion tool at DAVID Bioinformatics Resources 6.7.

### Association between Changes in Gene Expression and DNA Methylation Induced by the Treatment

Odds ratios (OR) were calculated to quantify the association between changes in gene expression and DNA methylation. Methylation and gene expression changes were represented as binary variables and the odd ratios were computed according to the formula: OR = (a/c)/(b/d). Where “a” is the number of genes differentially methylated and differentially expressed; “b” is the number of genes differentially methylated with not changes in gene expression; “c” is the number of genes with not changes in methylation but differentially expressed; “d” is the number of genes that did not changed their methylation or gene expression levels. Genes were classified as differentially methylated or not according to whether or not they contained a DMS in the sequence flanking their TSS (−3 kbp to 2 kbp).

### Creation of Simulated Dataset Containing Genomic Coordinates for Random Selected CG Sites

We calculated an empirical p-value to measure the strength of evidence provided by Figure 4 for suggesting an enrichment of DMS in the vicinity of the TSS. The estimated p value was obtained with the formula p = (*e* +1)/(*s* +1), where *s* is the number of datasets that have been simulated and *e* is the number of these dataset whose relative distribution of DMS, in the ±500 bp interval around the TSS, are smaller than or equal to that calculated including all surveyed CG sites.

To simulate these datasets, the distance between a given CG position and the proximal TSS was calculated for each one of the 634,009 CG sites included in this study. In each simulation a random number, ranging from 1 to 650,000 was assigned to each of these CG sites. Random numbers were returned using the formula = RAND()*(650,000-1)+1 in an excel spreadsheet. Cells in both columns were sorted together. The first ***n*** CG sites (ranked according to number randomly assigned) were considered DMS and represent one of the 100 simulated data set, where ***n*** is the number of DMS at FDR <0.01 calculated from the actual data (Dataset S2).

## Supporting Information

Figure S1 **A**, The total number of CG dinucleotides (row 1), the number of CGs included in the recognition sequences of any of the 4 enzymes used in this study (row 2) and the ratio of CGs that can be sampled using the four restriction enzymes (row 3); **B**, The Arabidopsis genome has been in-silico-fragmented into segments with randomly determined lengths. For each fragment the number of CG were counted (X axes) and the number of restriction sites used in this study were counted (Y axes); C, distribution of CG or surveyed CG (inside restriction sites) in different genome compartments.
(PNG)

Data Set S1 **MSCC alignments results expressed as number of reads per addressed CG.**
(ZIP)

Data Set S2 **MSCC t test results.**
(TXT)

Data Set S3 **Analysis of differentially expressed genes.**
(XLSX)

Data Set S4 **Gene Ontology enrichment analysis of the differentially expressed gene lists.**
(TXT)

Data Set S5 **Lists of genes used for Gene Ontology enrichment analysis.**
(TXT)

Data Set S6 **PEG treatment protocol; Structure of the DNA molecules in the CG tag libraries; Summary of the raw sequencing data.**
(PDF)

File S1 **Keys to the column headings in the data set S2, S3, S4 and S5.**
(PDF)
