# Supplementary figures and images for: Genome-Wide Quantitative Identification of DNA Differentially Methylated Sites in Arabidopsis Seedlings Growing at Different Water Potential
Source: PLoS One. 2013 Apr 8;8(4):e59878. doi: 10.1371/journal.pone.0059878 (PMC3620116; doi:10.1371/journal.pone.0059878)

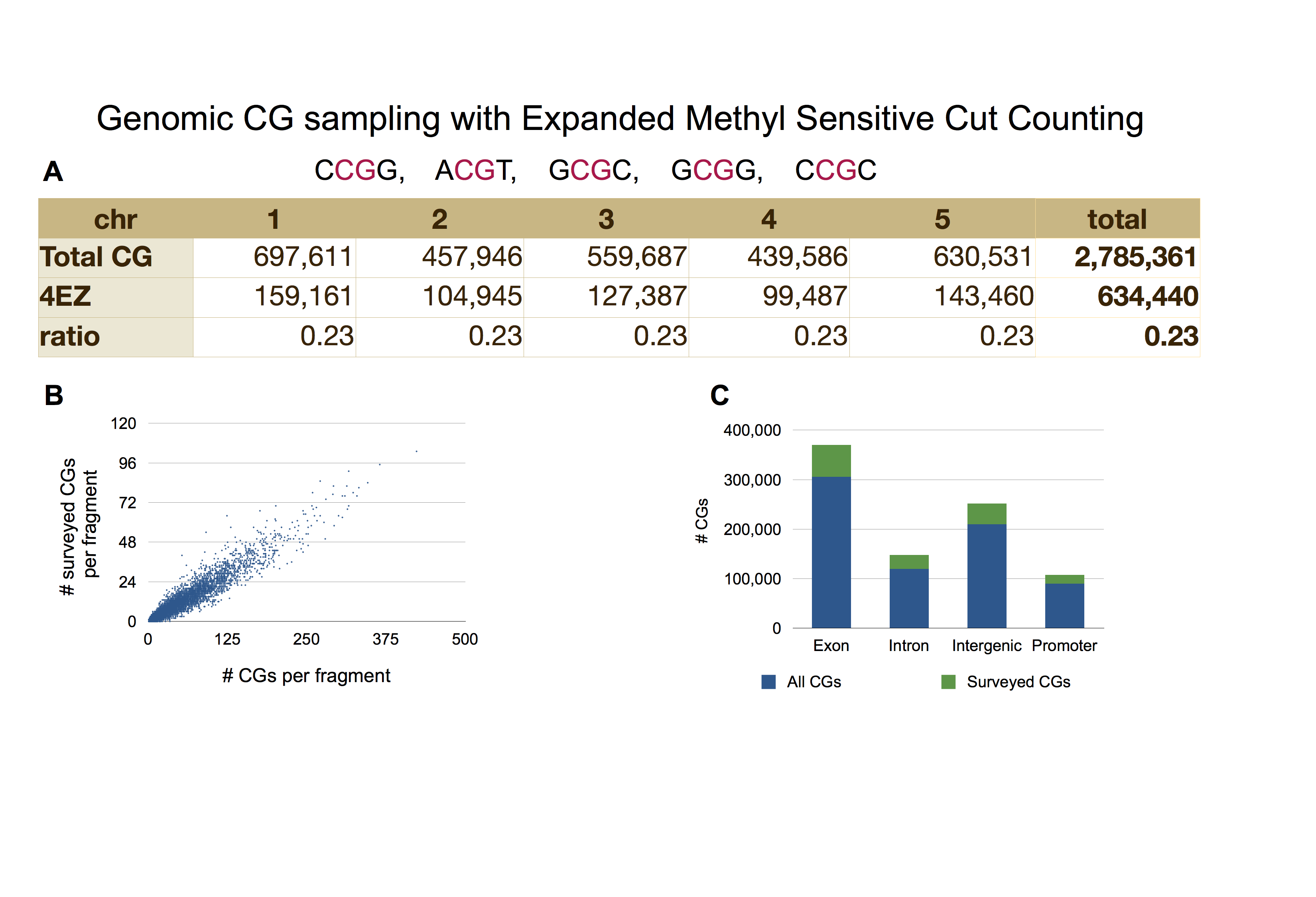

Supplement: Figure S1 — A, The total number of CG dinucleotides (row 1), the number of CGs included in the recognition sequences of any of the 4 enzymes used in this study (row 2) and the ratio of CGs that can be sampled using the four restriction enzymes (row 3); B, The Arabidopsis genome has been in-silico-fragmented into segments with randomly determined lengths. For each fragment the number of CG were counted (X axes) and the number of restriction sites used in this study were counted (Y axes); C, distribution of CG or surveyed CG (inside restriction sites) in different genome compartments. (PNG) [file pone.0059878.s001.png]
