# Supplementary material for: Genome-Wide Quantitative Identification of DNA Differentially Methylated Sites in Arabidopsis Seedlings Growing at Different Water Potential
Source: PLoS One. 2013 Apr 8;8(4):e59878. doi: 10.1371/journal.pone.0059878 (PMC3620116; doi:10.1371/journal.pone.0059878)

PEG TREATMENT PROTOCOL

1. **Cut paper filter paper in ~12 cm × 12 cm pieces** (Chromatography paper, Fisherbrand Cat# 05-714-4)) to cover the 12 cm × 12 cm square plates (Greiner petri dishes, Sigma-Aldrich, Cat# Z617679-240EA). Wrap the pieces of paper with aluminum foil and autoclave.
2. **Prepare one-quarter MS, 2 mM MES media plates**. For one set of 24 plates, 1 L of media containing 1.1 g of MS salts and 0.4 g of MES (adjusted to pH 5.7 using KOH) and 15 g of agar will be needed. Autoclave the media. Pour 24 agar plates or as much as possible and store to 4 °C if not immediately used.
3. Immediately before sow seeds in the above plates, **place a piece of sterilized paper on each plate** and wait to the papers get completely wet.
4. Aliquot and **sterilize an appropriate amount of seed**. Approximately 100–150 seeds will be needed for each plate and typically 10 plates are prepared for each genotype-condition combination being tested.
5. Put seeds in a 2 ml eppendorf tube, add 1 ml of 70% ethanol and mix constantly for 10 min. Discard the 70% ethanol and add 1 ml of 95% ethanol and mix constantly for another 10 min. Pour the suspension in a sterile petri dish containing a piece of sterile paper filter. Try to spread the seeds on the surface of the filter. **Let seeds dry; usually will take 20 to 30 min**.
6. After the seeds are sterilized, **use a sterile toothpick to place seed on the plates**. Take care that the seed is spread out well so that the seedlings grow mostly in a single layer and not in clumps**.** Use micro-pore tape (1 in width, 3M 1530-1) to secure the plate lids.
7. **Stratify at 4 °C for 3 days** and then transfer the plates to a growth chamber. **Incubate plates vertically in a growth chamber for 7 days.** Typical growth conditions are 23°C with continuous light (80–100 $\mu mol/m^2/s$).

Prepare PEG–agar plates:

8. Prepare 1 L of media with agar as described in **step 2** and adjust pH to 5.7. Prepare a second liter of media with the MS salts and MES buffer but without adding agar (liquid media). Aliquot the liquid media into 1L plastic beakers (400 ml aliquots). Add a stir bar to each beaker; make a lid with aluminum foil and autoclave everything.
9. From the autoclaved agar media, pour 40 mL of media into each of 24 of the 12 × 12 square Petri dishes and allow to cool. Note that this volume must be precisely controlled.
10. For the autoclaved liquid media, add 320 gr of PEG-8000 (poly ethylene

glycol, Sigma Aldrich Cat# P5413) to each beaker. Stir to dissolve the PEG (best done when media is still hot). After the media has cooled, adjust the pH to 5.7 with 20% HCl, (3 to 5 drops must be necessary depending on the transfer pipet) .

11. **Transfer 60 mL of PEG solution onto the solidified agar of each plate** (an autoclaved graduate cylinder with 100 ml capacity can be used). Prepare ten plates for each genotype tested. Pile the plates making stacks of 10 units, put a weight on top of each stack (a one litter bottle full of water can be used), and **incubate for at least 12 h before use**.
12. **When seedlings have grown for 7 days, transfer them to the PEG–agar plates.** Pour off as much PEG as possible and use forceps to lift the paper containing 7 day old seedlings off their original plate; place the paper on the PEG plate and make sure that all parts of it are in contact with the PEG–agar media. Seal the plates with micro-pore tape and **place plates back into the growth chamber, again in the vertical position, for an additional 3 days.**

Structure of the DNA molecules in the CG tag libraries with detail of the oligos used during the libraries preparation. First and second adaptors are indicated in black, the CG tag genomic sequence is indicated in green, the primers used to amplify the libraries are indicated in blue and the primer used to sequence the libraries is indicated in red.

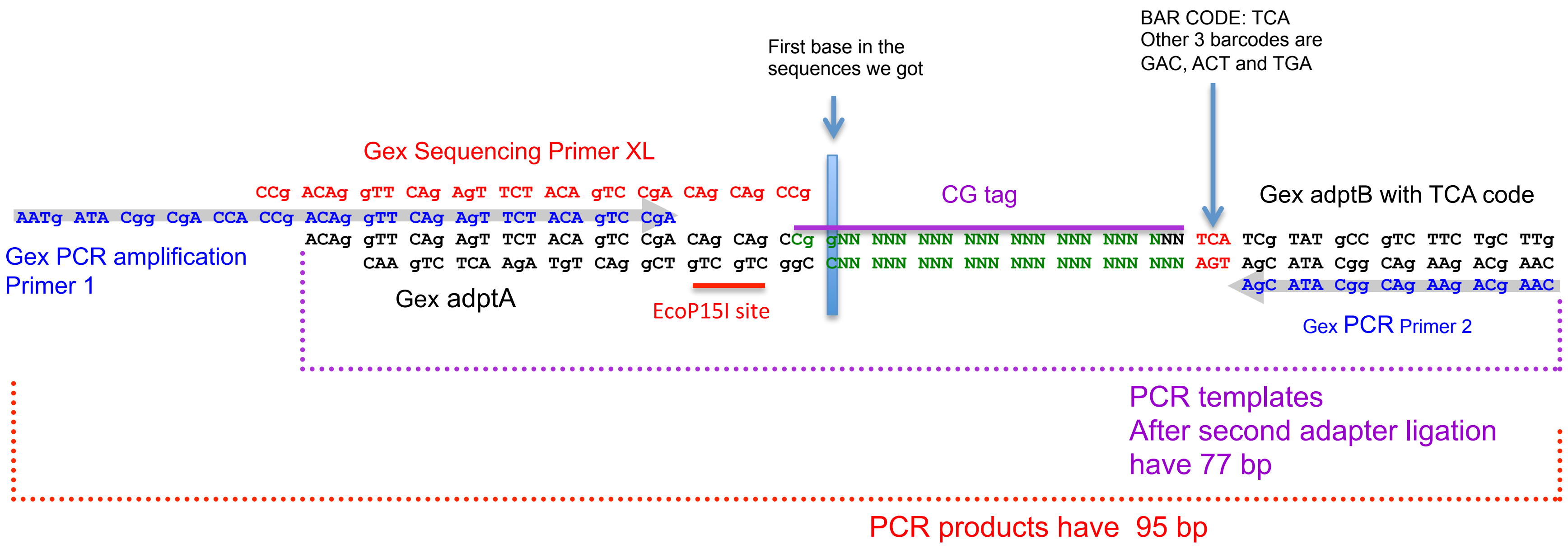

Number of aligned reads per library

Control

-0.25 MPa

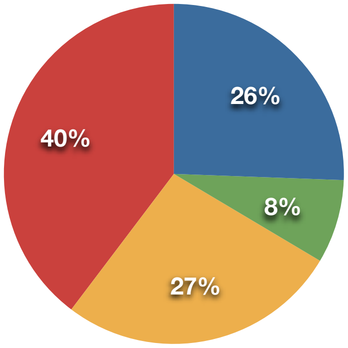

Rep. 1	s3ACT	7,545,622 reads
Rep. 2	s3GAC	2,346,008 reads
Rep. 3	s3TCA	7,896,101 reads
Rep. 4	s3TGA	11,706,868 reads

Drought

-2 MPa

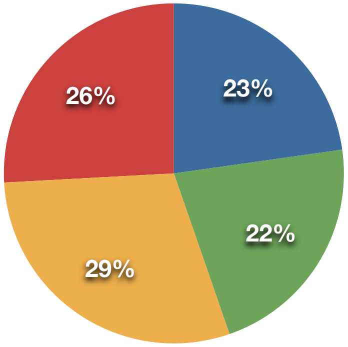

Rep. 1	s4ACT	7,529,743 reads
Rep. 2	s4GAC	7,251,810 reads
Rep. 3	s4TCA	9,745,127 reads
Rep. 4	s4TGA	8,554,296 reads

Key to headers describing data contained in columns N, O, Q and S in Data Set S2 Example: DMS in gene ATCSLD5 (chr1:598,295)

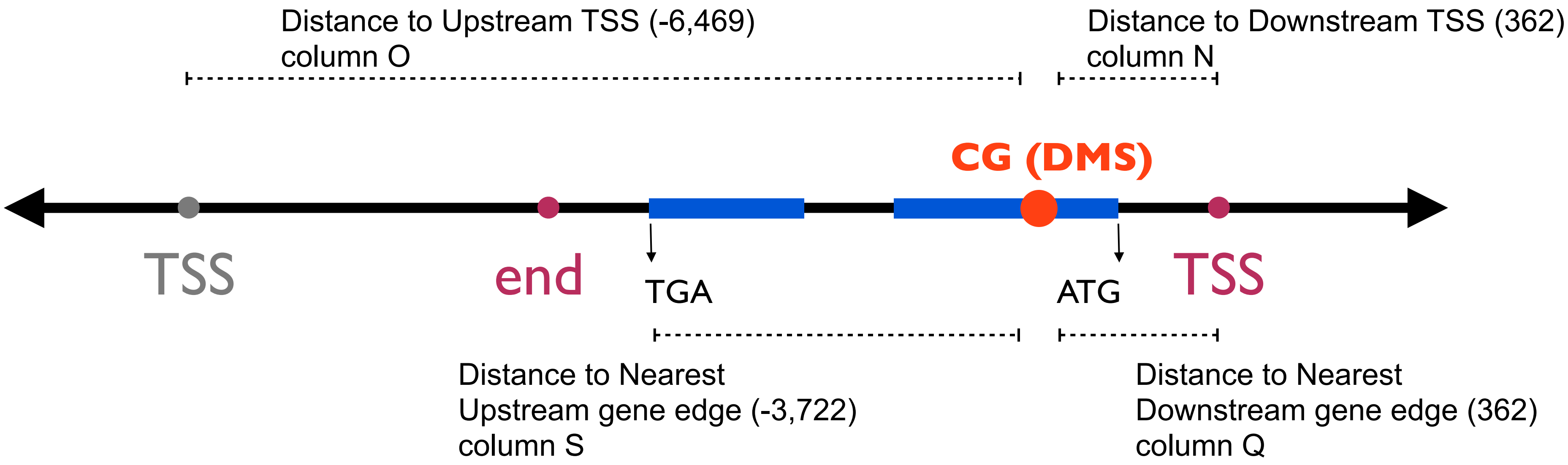

Supplement: Data Set S6 — PEG treatment protocol; Structure of the DNA molecules in the CG tag libraries; Summary of the raw sequencing data. (PDF) [file pone.0059878.s007.pdf]
