# Supplementary material for: Genome-Wide Quantitative Identification of DNA Differentially Methylated Sites in Arabidopsis Seedlings Growing at Different Water Potential
Source: PLoS One. 2013 Apr 8;8(4):e59878. doi: 10.1371/journal.pone.0059878 (PMC3620116; doi:10.1371/journal.pone.0059878)
Supplement: File S1 — Keys to the column headings in the data set S2, S3, S4 and S5. (PDF) [file pone.0059878.s008.pdf]

**Data Set S2 MSCC t test results**		
**Column #**	**Header**	**Description**
1 or A	Chr(1 to 5)	Identity of the chromosome containing the restriction site described in the row
2 or B	pos	Position of the chromosome containing the restriction site described in the row
3 or C	S3ACT	*Digestion frequencies* scored for the restriction site described in the row. The value represents the number of sequences aligned in this position of the chromosome. The results correspond to the **first replicate** of the control-samples (plants grown on medium **without PEG)**.
4 or D	S3GAC	Id. to previous column, but **second replicate without PEG**.
5 or E	S3TCA	Id. to previous column, but **third replicate without PEG**.
6 or F	S3TGA	Id. to previous column, but **fourth replicate without PEG**.
7 or G	S4ACT	*Digestion frequencies* scored for the restriction site described in the row. The value represents the number of sequences aligned in this position of the chromosome. The results correspond to the **first replicate** of the treated-samples (plants grown on medium **infused with PEG)**.
8 or H	S4GAC	Id. to previous column, but **second replicate in PEG**.
9 or I	S4TCA	Id. to previous column, but **third replicate in PEG**.
10 or J	S4TGA	Id. to previous column, but **fourth replicate in PEG**.
11 or K	Pass FDR 0.05?	Results of the t test applied to the *digestion frequencies* when the false discovery rate (**FDR**) is controlled below **0.05**. If the averages of the replicates for samples **with-PEG** and **without-PEG** result **different**, then the cells contain the word "**yes**". Otherwise contain the word "no".
12 or L	Pass FDR 0.01?	Results of the t test applied to the *digestion frequencies* when the false discovery rate (**FDR**) is controlled below **0.01**. If the averages of the replicates for samples **with-PEG** and **without-PEG** result **different**, then the cells contain the word "**yes**". Otherwise contain the word "no".
13 or M	Unmet or Met?	After moving the plants to a medium with a water potential of -2MPa, the restriction site described in the row could: acquire methylation **"M"**, lose methylation **"U"** or remain unchanged **"-"**. These calls are based on the results described in column 11 or K.
14 or N	Distance to Downstream TSS	The value in each cell represents the distance from the restriction site described in the row to the nearest downstream transcription initiation site (TSS). See figure in Data Set S6, page 6 for a graphic description.
15 or O	Distance to Upstream TSS	The value in each cell represents the distance from the restriction site described in this row to the nearest upstream transcription initiation site (TSS). See figure in Data Set S6, page 6 for a graphic description.
16 or P	Nearest Upstream gene	Each cell contains the official gene symbol of the gene overlapping or located upstream of the restriction site described in the row. See figure in Data Set S6, page 6 for a graphic description.
17 or Q	Distance to Nearest Downstream gene edge	The value in each cell represents the distance from the restriction site described in the row to the nearest upstream gene edge. See figure in Data Set S6, page 6 for a graphic description.
18 or R	Nearest Downstream gene	Each cell contains the official gene symbol of the gene overlapping or located downstream of the restriction site described in the row. See figure in Data Set S6, page 6 for a graphic description.
19 or T	Between gene edges?	If the restriction site described in the row is located **between** the **start and stop codons** of a gene, then the cells contain the word "**yes**". Otherwise contain the word "no".
20 or U	in Exon_1?	If the restriction site described in the row is located in exon 1 of a gene, then the cells contain the word "**yes**". Otherwise contain the word "no".
21 or V	In_other Exon?	If the restriction site described in the row is located in an exon different from exon 1, then the cells contain the word "**yes**". Otherwise contain the word "no".
22 or W	In_Intron	If the restriction site described in the row is located in an intron, then the cells contain the word "**yes**". Otherwise contain the word "no".

Data Set S3 analysis of differentially expressed genes		
**Column #**	**Header**	**Description**
1 or A	Gene Symbol	AGI code
2 or B	p-value (-0.25 MPa and -2 MPa)	The p-value for the linear contrast of the two levels (-0.25 MPa and -2 MPa) within the factor (3 days treatment).
3 or C	MeanRatio (-0.25 MPa and -2 MPa)	The ratio of the means (RPKMs from four replicates) of the two groups (-0.25 MPa and -2 MPa). Group -0.25 MPa is the reference and constitute the denominator of the ratio. Numbers less than one imply down-regulation
4 or D	FoldChange (-0.25 MPa and -2 MPa)	Linear fold change, where a value of “0” indicates “NO” change, positive values indicate the gene is up-regulated in plants growing at -2MPa. Negative values indicate the opposite.

Data Set S4 Gene Ontology enrichment analysis of the differentially expressed gene lists		
**Column #**	**Header**	**Description**
1 or A	GO Term Description	GO term name in the GO database.
2 or B	p-value	Is the p-value associated to the computed enrichment in column 4 or D.
3 or C	FDR q-value	Is the correction of the above p-value for multiple testing (3967 GO terms) using the Benjamini and Hochberg method.
4 or D	Enrichment (N, B, n, b)	The first number represent the enrichment computed with the formula: Enrichment = (b/n) / (B/N). The numbers inside the bracket represent: total number of Arabidopsis genes associated with any GO term (**N**); total number of genes associated with a specific GO term (**B**); number of genes in the up or down regulated gene list (**n**); number of genes in the intersection (**b**).

Data Set S5 lists of genes used for Gene Ontology enrichment analysis		
**Column #**	**Header**	**Description**
1 or A	All Arabidopsis genes AGI codes	This column contains all the genes used as background to test for GO terms enrichment in the target list of genes (columns 3 to 6)
2 or B	All Arabidopsis genes OFFICIAL GENE SYMBOL	Each cell contains the **official gene symbol** for the gene described in the cell located in same row of the previous column (column 1 or A).
3 or C	Genes with CG methylation in their promoters	Each cell contains the official gene symbol for the genes in the target list
4 or D	Genes with CG demethylation in their promoters	Each cell contains the official gene symbol for the genes in the target list
5 or E	Down Regulated genes	Each cell contains the official gene symbol for the genes in the target list
6 or F	Up regulated genes	Each cell contains the official gene symbol for the genes in the target list

To reproduce the analysis made in the present manuscript follow the link bellow.

http://cbl-gorilla.cs.technion.ac.il/

- Once in the website, follow Step 1 to select Arabidopsis as the organism.
- Proceed to Step 2 and select: Two unranked list of genes (target and background lists)
- Proceed to Step 3 and copy-paste any of the target lists from Data set S5 in the “Target set” window. Copy-paste data from the column 2 in Data set S5 to the “Background set” window.
- Proceed to Step 4 and “Choose an ontology”, for example “All”
- We have selected P-value threshold to a minimum of 10^-4
- Click on “Search Enriched GO terms” to proceed to the results page.
- Results are shown in the form of a *directed acyclic graph* (DAG). All terms shown on the screen were significantly enriched at the selected p-value. By clicking on any of the boxes you can access to the genes in the target lists associated with the selected GO term.
